# Ameliorating Effects of *Phlomis umbrosa* Turcz. Root in Ovalbumin-Induced Allergic Asthma: Modulation of IL-33-Mediated Inflammation and TGF-β/Smad-Dependent Fibrosis

**DOI:** 10.3390/antiox15040420

**Published:** 2026-03-27

**Authors:** Yeong Hyeon Ju, Hyo Lim Lee, Hye Ji Choi, Yu Mi Heo, Hwa Rang Na, Ho Jin Heo

**Affiliations:** Division of Applied Life Science (BK21), Institute of Agriculture and Life Science, Gyeongsang National University, Jinju 52828, Republic of Korea; ju8172001@gnu.ac.kr (Y.H.J.); gyfla059@gnu.ac.kr (H.L.L.); hjchoi0820@gnu.ac.kr (H.J.C.); yumi@gnu.ac.kr (Y.M.H.); hrna@gnu.ac.kr (H.R.N.)

**Keywords:** *Phlomis umbrosa* Turcz., allergic asthma, Th2 cytokine, IL-33, TGF-β signaling

## Abstract

Our study aimed to evaluate the therapeutic potential of a 20% ethanolic extract of the *Phlomis umbrosa* Turcz. (EPT) herb and its associated bioactive compounds in an ovalbumin (OVA)-induced allergic asthma mouse model. We used phytochemical analysis and identified sesamoside, shanzhiside methyl ester, 8-O-acetyl shanzhiside methyl ester, and isoacteoside as the bioactive components. We validated and quantitatively analyzed shanzhiside methyl ester as a major compound. The treatment with EPT significantly attenuated the T helper type 2 (Th2)-based immune response, eosinophilia, histopathological changes, and biochemical parameters. Furthermore, EPT inhibited interleukin (IL)-33-mediated activation of the nuclear factor kappa B (NF-κB) and transforming growth factor beta (TGF-β) signaling pathways, as well as reduced fibrosis and apoptosis associated with inflammation. The findings of our study suggest that EPT is a promising natural substance for alleviating symptoms of allergic asthma.

## 1. Introduction

Asthma affects more than 350 million individuals worldwide, with a steadily increasing global prevalence [1]. It is a chronic inflammatory disease characterized by bronchial hyperresponsiveness, mucus hypersecretion, airway inflammation, and subsequent airway remodeling [2]. The airway becomes stiffened and narrowed due to these pathological changes, resulting in partially reversible expiratory airflow limitation that clinically manifests as dyspnea, wheezing, cough, and chest tightness [3].

Asthma is broadly categorized into allergic and non-allergic phenotypes based on the type of trigger [4]. Allergic asthma, the most prevalent type, arises from an exaggerated immune response to environmental allergens, including house dust mites, pollen, pollution, and dietary components [5]. This response is driven by T helper type 2 (Th2) cell-mediated inflammatory pathways [6]. Inhaled allergens damage airway epithelial cells, triggering the release of alarmins, such as interleukin (IL)-33, which activates antigen-presenting dendritic cells (DCs) [4]. These activated DCs bind allergens, migrate to the lymph nodes, and drive naïve CD4^+^ T cell differentiation toward the Th2 lineage [7]. Subsequently, Th2 cells circulate to the lungs, secrete considerable amounts of Th2 cytokines, including IL-4, IL-5, and IL-13, which stimulate B cells to produce immunoglobulin E (IgE) and promote the infiltration and activation of inflammatory cells [7]. Activated inflammatory cells, including eosinophils, lymphocytes, and monocytes, infiltrate lung tissues and release inflammatory mediators that contribute to chronic airway inflammation [8].

At injury sites, recruited inflammatory cells execute respiratory bursts for phagocytosis, depleting oxygen rapidly and generating excessive reactive oxygen species (ROS) [9]. High amounts of ROS lead to an imbalance between endogenous antioxidant systems, such as reduced glutathione (GSH) and superoxide dismutase (SOD), and oxidants, resulting in increased oxidative stress in lung tissues [10]. This oxidative stress activates transcription factors such as c-Jun N-terminal kinase (JNK) and nuclear factor kappa B (NF-κB) through IL-33-mediated pathways [9]. This creates a vicious cycle that promotes inflammatory pathways [11]. Furthermore, alveolar macrophages contribute to subepithelial fibrosis by secreting transforming growth factor beta (TGF-β) via the TGF-β/suppressor of mothers against the decapentaplegic (Smad) pathway in the inflammatory environment [12]. This results in airway remodeling with decreased lung function and progressively worsening asthma [3].

Currently, drugs primarily used to treat allergic asthma, including long-acting β2-agonists, inhaled corticosteroids, and leukotriene modifiers, primarily focus on symptom control [2]. However, these drugs are reported to exhibit significant interindividual differences in treatment response due to differences in disease characteristics, genetic factors, and biomarker profiles, which limit their therapeutic effectiveness [10,13]. In addition, prolonged or repeated administration of these drugs is associated with reduced therapeutic responsiveness, such as tachyphylaxis, as well as an increased risk of serious systemic adverse effects [14]. In particular, long-term use of oral corticosteroids carries the risk of various side effects, including gastrointestinal symptoms, hypertension, osteoporosis, metabolic disorders, cardiovascular disease, and adrenal suppression [15]. For these reasons, existing drugs may be effective in alleviating symptoms but may not provide sufficient therapeutic benefit in patients with severe asthma and allergic diseases [13]. Accordingly, biological therapeutics targeting specific cytokines, immune cells, and signaling pathways that induce allergic inflammation have been developed recently [2]. Biologic agents such as omalizumab, mepolizumab, reslizumab, and dupilumab, which target IgE, IL-5, and IL-4Rα signaling pathways, have contributed to improved disease control in patients with severe asthma [15]. However, their high cost and limited accessibility still limit their widespread clinical application [15]. Therefore, the need for the development of safer and more effective asthma management strategies is continuously raised [6]. Against this backdrop, recent research is investigating natural product-derived candidate substances with anti-inflammatory activity that can modulate the inflammatory response in asthma [6,10]. For example, tomato juice and extracts have been reported to suppress airway inflammatory responses, and genistein, a soy isoflavone, has also been reported to alleviate eosinophilic inflammation and asthma severity by inhibiting eosinophil leukotriene C4 synthesis and the MAPK signaling pathway [10]. These findings suggest that various plant-derived natural compounds with anti-inflammatory activity may be promising candidates for asthma management.

Belonging to the Lamiaceae family, *Phlomis umbrosa* Turcz. (*P. umbrosa*) is a perennial herb native to Korea and northern China [16]. *P. umbrosa* is used as a traditional herbal medicine to treat inflammation, pain, and phlegm by relieving edema, as described in the *Dongui Bogam* [17]. The biological properties of *P. umbrosa* are attributed to its diverse phytochemical constituents, including flavonoids, phenolic compounds, iridoid, and phenylethanoid glycosides, such as 8-O-acetyl shanzhiside methyl ester, shanzhiside methyl ester, and sesamoside [18,19]. Owing to these bioactive constituents, *P. umbrosa* reportedly exhibits multiple biological effects, such as anti-inflammatory, and antioxidant activities [18]. Pak et al. [17] demonstrated, through network pharmacology analysis in *P. umbrosa*, a close association between the mitogen-activated protein kinase (MAPK) signaling pathway and an asthma-related pathway. This association was verified through ex vivo experiments that suggested the anti-asthmatic potential of *P. umbrosa* [17]. However, such studies were primarily confined to inflammatory responses. There is limited research that verifies the initial mechanisms of fibrosis mediated by the TGF-β1/Smad signaling pathway. Therefore, our study aimed to identify the main bioactive compounds of the 20% ethanolic extract of *P. umbrosa* (EPT) and to evaluate its anti-inflammatory and antifibrotic efficacy on Th2-mediated inflammation and the TGF-β1/Smad pathways in an ovalbumin (OVA)-induced allergic asthma mouse model. In addition, the physicochemical and pharmacokinetic properties of the identified compounds were assessed through in silico absorption, distribution, metabolism, and excretion (ADME) prediction to evaluate their suitability as bioactive natural product leads with potential functional applications.

## 2. Materials and Methods

### 2.1. Chemicals and Reagents

Acetonitrile and methanol, solvents used in high-performance liquid chromatography (HPLC)-grade solvents for HPLC and ultra-performance liquid chromatography-quadrupole time-of-flight tandem-mass spectrometry (UPLC-QTOF-MS/MS) were HPLC-grade solvents and purchased from Thermo Fisher Scientific (Waltham, MA, USA). Shanzhiside methyl ester (cat. no. BF-S1010), used as an analytical standard, was obtained from Biofron (La Mirada, CA, USA). For the animal model, OVA (cat. no. A5503) and aluminum hydroxide (cat. no. 239186) were sourced from Sigma-Aldrich (St. Louis, MO, USA).

### 2.2. Plant Material and Extraction of P. umbrosa

The dried roots of *P. umbrosa* used in this study were purchased from Jayeon Chunsa Co., Ltd., located in Jecheon, North Chungcheong Province, Republic of Korea, on 3 January 2023. The plant material was collected from Jecheon (37.132582, 128.190948) in October 2022. The roots of *P. umbrosa* were finely ground and extracted under reflux with 20% ethanol (1:50, *w*/*v*) for two h at 40 °C. Following filtration and vacuum concentration, the extract was lyophilized and stored at −20 °C until analysis.

### 2.3. UPLC-QTOF-MS/MS

EPT was reconstituted at 2 mg/mL in 50% methanol and then passed through a 0.45 μm syringe filter (Whatman, Kent, UK). Identification of bioactive compounds was performed using a Nexera XS UPLC system (Shimadzu, Kyoto, Japan), interfaced with an X500R QTOF system (AB SCIEX, Seoul, Republic of Korea), and fitted with an Acquity UPLC BEH C_18_ column (2.1 mm × 100 mm, 1.7 μm; Waters, Milford, MA, USA) at the High-Tech Materials Analysis Core Facility (Gyeongsang National University, Jinju, Republic of Korea). A gradient system was used for chromatographic separation with mobile phase A (0.1% formic acid in distilled water) and mobile phase B (0.1% formic acid in acetonitrile) at a flow rate of 0.35 mL/min. Gradient conditions were programmed as follows: 0 to 18 min, 0% to 80% B; 18 to 20 min, 80% to 0% B; 20 to 25 min, held at 0% B. Mass spectrometric conditions were optimized under electrospray ionization negative ion mode. The ion source and desolvation temperature were maintained at 100 °C and 400 °C, respectively, with a capillary voltage of 2.5 kV. Collision energy ranged from 20 to 50 V, and full-scan acquisition covered the mass-to-charge ratio (*m*/*z*) range of 50–1500. The 2D structures of the identified bioactive compounds were generated using the MolDraw program (https://www.moldraw.com/, accessed on 11 March 2026).

### 2.4. HPLC with Photodiode Array (HPLC-PDA) Detection

EPT was reconstituted at 3 mg/mL in 50% methanol and filtered through a 0.45 μm syringe filter (Whatman). Quantitative analysis and method validation were performed using an HPLC system (Ultramate 3000 series, Thermo Fisher Scientific), coupled with a PDA. Chromatographic separation was performed on a YMC-Triart BEH C_18_ column (4.6 mm × 150 mm, 5 μm; YMC, Seongnam, Republic of Korea) maintained at 40 °C. A gradient system was used for chromatographic separation with mobile phase A (0.1% formic acid in distilled water) and mobile phase B (0.1% formic acid in methanol) at a flow rate of 1 mL/min. Gradient conditions were programmed as follows: 0 to 2 min, 5% B; 2 to 12 min, 5% to 15% B; 12 to 22 min, 15% to 50% B; 22 to 25 min, 50% B; 25 to 30 min, 50% to 100% B; 30 to 32 min, 100% to 5% B. Chromatograms were recorded at 238 nm.

### 2.5. Validation of HPLC Analysis Method

The HPLC method for quantifying shanzhiside methyl ester in EPT underwent validation according to Association of Official Analytical Chemists (AOAC) guidelines for standard method performance requirements [20]. Validation parameters included specificity, linearity, sensitivity, precision, and accuracy.

#### 2.5.1. Specificity

Specificity was assessed by comparing the retention time (RT) and spectral characteristics of the shanzhiside methyl ester standard with the corresponding peak detected in EPT. Peak identity confirmation was performed using Chromeleon^TM^ Chromatography Data System Software (version 6.8, Thermo Fisher Scientific) through spectral matching analysis.

#### 2.5.2. Linearity

Linearity was determined by constructing calibration curves based on five standard concentrations of shanzhiside methyl ester (1, 2, 5, 10, 20, and 50 μg/mL). The relationship between the peak area and concentration was analyzed by linear regression, and the correlation coefficient (R^2^) was used to confirm linearity.

#### 2.5.3. Quantification

Quantification of shanzhiside methyl ester in EPT was performed using a calibration curve constructed as mentioned above. The content was determined by interpolating the peak area corresponding to shanzhiside methyl ester from the EPT chromatogram into the calibration curve.

#### 2.5.4. Sensitivity

Sensitivity parameters, such as the limit of detection (LOD) and limit of quantification (LOQ), were calculated from the shanzhiside methyl ester calibration curve. LOD was calculated from 3.3 × σ/S, and LOQ was obtained from 10 × σ/S, where σ denotes the standard deviation of the y-intercept, and S represents the slope of the regression equation.

#### 2.5.5. Precision

Precision was assessed through repeatability and reproducibility analyses by repeatedly injecting a 20 μg/mL standard solution of shanzhiside methyl ester. Repeatability was evaluated using five replicate injections within a single day, while reproducibility was assessed by performing three injections over three consecutive days, reflecting intra-day and inter-day variability, respectively. The results were expressed as relative standard deviation (RSD, %).

#### 2.5.6. Accuracy

Accuracy was determined through spike-recovery tests. Known quantities of shanzhiside methyl ester (27.2, 34, and 40.8 μg/mL) were added to the EPT solution to yield final concentrations equivalent to 80%, 100%, and 120% of the expected amount. The recovery percentage was calculated using the following formula:

$$Recovery\;(\%)=\;\frac{Measured\;amount\;after\;spiking-Original\;amount}{Added\;amount}\times 100$$

### 2.6. Animals, Sensitization, and Treatment

**Figure 1:**
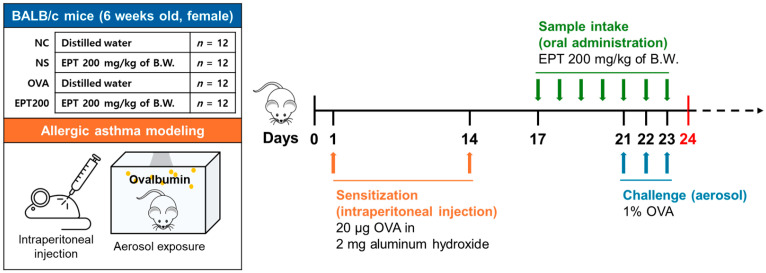
Schematic diagram for the ovalbumin (OVA)-induced allergic asthma model.

Six-week-old BALB/c female mice were purchased from Koatech (Pyeongtaek, Republic of Korea). All animals were acclimated and maintained in an environment with a temperature of 22 ± 1 °C, humidity of 50 ± 5%, and a 12 h light cycle starting at 06:00. Standard rodent chow and drinking water were available ad libitum throughout the experimental period. The animals were randomly allocated into four experimental groups of twelve mice each according to treatment: normal control (NC), normal sample (NS) (EPT 200 mg/kg of body weight (B.W.)), OVA-sensitized (OVA), and OVA-sensitized with EPT (EPT200, EPT 200 mg/kg of B.W.) treatment. Twelve mice per group were allocated to different experimental analyses before the study began: (a) five mice per group for biochemical analysis (fluorescence-activated cell sorting (FACS), IgE, white blood cells (WBCs) differential counting, and antioxidant parameters analysis), (b) three mice per group for histological examination, and (c) four mice per group for Western blot analysis. The construction of the OVA mice model was established according to the following procedure (Figure 1). Briefly, mice received two intraperitoneal sensitizations (days 1 and 14) with 20 μg OVA emulsified in 2 mg aluminum hydroxide in 200 μL phosphate-buffered saline (PBS), followed by three consecutive aerosol challenges (days 21–23) with 1% OVA solution for 30 min daily using a nebulizer (Omron, Kyoto, Japan). Normal animals (NC and NS groups) were exposed to PBS aerosol. During the intervention period (days 17–23), NC and OVA groups received distilled water by oral gavage, while NS and EPT200 groups received EPT. Here, EPT200 refers to the OVA-sensitized mice treated with 200 mg/kg EPT. On day 24, all animals were euthanized, and blood, bronchoalveolar lavage fluid (BALF), and lung tissues were extracted. All animal experiments were performed in accordance with the ARRIVE guidelines to ensure ethical and scientific validity and to safeguard animal welfare. The experimental protocols were reviewed and approved by the Institutional Animal Care and Use Committee of Gyeongsang National University (Approval No. GNU-240405-M0076; 5 April 2024). All procedures complied with relevant institutional, national, and international regulations, including EU Directive 2010/63/EU.

### 2.7. FACS Analysis

The collected whole blood from the abdominal vena cava was labeled with BV786 anti-CD3e, APC anti-CD4, and PerCP-Cy5.5 anti-CD8a for 30 min at 4 °C. Subsequently, erythrocytes were removed using a lysing solution, and the pellet was rinsed with staining buffer. The remaining cells were permeabilized and fixed by reacting with the fixation/permeabilization solution for 20 min at 4 °C. Intracellular cytokines were labeled with BV421 anti-interferon (IFN)-γ and PE anti-IL-4 for 30 min at 4 °C, followed by rinsing with the wash buffer. Cells were resuspended in the staining buffer and analyzed using the BD FACSLyric^TM^ Clinical Cell Analyzer (BD Bioscience, Franklin Lakes, NJ, USA). Data acquisition and analysis were conducted using FlowJo (version 10.10.0, BD Biosciences). Detailed information on antibodies is provided in Table S1.

### 2.8. Measurement of OVA-Specific IgE Levels

Serum was obtained by centrifuging whole blood at 10,000× *g* for 15 min at 4 °C. In addition, the trachea was partially excised, an intravenous catheter was inserted into the airway, and the BALF was harvested after 0.7 mL of PBS was gently perfused from the airway to the lung, a process repeated twice. The OVA-specific IgE levels in the serum and BALF were quantified using the mouse OVA-specific IgE kit (BioLegend, San Diego, CA, USA).

### 2.9. Western Blot Analysis

Lung tissues were prepared under the conditions described in Lee et al. [21], electrophoresed onto an SDS-polyacrylamide gel, and transferred onto a PVDF membrane (Millipore, Burlington, MA, USA). Membranes were blocked with 5% skim milk for one h at room temperature. Subsequently, the membranes were reacted with the primary antibodies overnight at 4 °C, followed by reaction with HRP-linked secondary antibodies for one h at room temperature. In the final step, the protein bands were visualized using an enhanced chemiluminescence reagent (TransLab, Daejeon, Republic of Korea) and captured with an iBright CL1500 Imaging System (Thermo Fisher Scientific). Densitometric quantification was performed using ImageJ (version 1.54d, National Institutes of Health, Bethesda, MD, USA). Detailed information on antibodies is provided in Table S1.

### 2.10. WBCs Differential Counting

After dissection, whole blood was collected from the abdominal vena cava into tubes containing ethylenediaminetetraacetic acid dipotassium salt dihydrate as an anticoagulant. BALF was prepared as mentioned above. Then, WBC counts, including neutrophils, lymphocytes, monocytes, and eosinophils, were analyzed using SYSMEX XN-V (Sysmex Corporation, Kobe, Japan).

### 2.11. Histopathological Analysis

The right lung tissues were dissected and immersed in 10% formalin for fixation. Following fixation, the tissues were embedded in paraffin, sectioned at a thickness of 4 μm, and stained with hematoxylin and eosin (H&E) for histological examination. High-resolution digital images were captured using a Motic EasyScan Pro 6 (Motic, Hong Kong, China). Histopathological evaluation was performed following the methodology of Chauhan & Singh [22], assessing peribronchiolar inflammatory cell infiltration, bronchiole wall thickness, and alveolar space. Peribronchiolar inflammation was scored on a 5-point scale: 0, normal; 1, few inflammatory cells; 2, one-layer ring; 3, two to four-layer ring; and 4, multi-layer ring (>4 layers) of inflammatory cells. Additionally, the thickness of the bronchiole wall was calculated as the airway wall thickness (μm) per length of basement membrane (mm), with measurements taken at four equidistant points (3, 6, 9, and 12 o’clock positions) per bronchiole. Also, the alveolar area was calculated by subtracting the area of epithelial cells from the total area (100%). Quantitative analysis of all histological images was conducted using ImageJ software (version 1.54d, National Institutes of Health, Bethesda, MD, USA).

### 2.12. Antioxidant Parameters

Lung tissues were prepared following the method described by Lee et al. [21]. The tissues were homogenized and centrifuged under the specified conditions. The obtained pellet and supernatant were collected for subsequent analysis of antioxidant parameters, including reduced GSH levels, SOD levels, and malondialdehyde (MDA) contents. Reduced GSH levels were expressed as a percentage of the NC group. SOD levels were expressed as U/mg protein based on a standard calibration curve using a SOD assay kit (Dojindo Molecular Technologies, Kumamoto, Japan). MDA contents were expressed as nM/mg of protein.

### 2.13. ADME-Based Physicochemical and Pharmacokinetic Profiling

The physicochemical and pharmacokinetic properties of the major phytochemicals identified in EPT were predicted using the web-based platform SwissADME (http://www.swissadme.ch, accessed on 10 March 2026). The canonical simplified molecular input line entry system (SMILES) of each compound was retrieved from the PubChem database (https://pubchem.ncbi.nlm.nih.gov, accessed on 10 March 2026) and used as structural inputs. Isoacteoside was excluded from this analysis because its SMILES string exceeded the character input limit of the SwissADME platform, rendering prediction unfeasible. The evaluated parameters included molar refractivity, which reflects molecular polarizability, and topological polar surface area (TPSA), which serves as an indicator of membrane permeability. Consensus Log P*_o_*_/_*_w_* was calculated to estimate lipophilicity, and gastrointestinal (GI) absorption was assessed to evaluate oral bioavailability potential. Blood–brain barrier (BBB) permeability and P-glycoprotein (P-gp) substrate status were examined to predict central nervous system accessibility and efflux transport liability. Inhibition of key cytochrome P450 (CYP450) isoforms, including CYP1A2, CYP2C19, CYP2C9, CYP2D6, and CYP3A4, was assessed to evaluate the potential for metabolic drug–drug interactions. Drug-likeness was further evaluated using Pan Assay Interference Compounds (PAINS) and Brenk filters to identify potentially problematic structural motifs. The physicochemical profiles of the compounds were visualized using the bioavailability radar charts generated by SwissADME, which display six key descriptors: lipophilicity (LIPO), size (SIZE), polarity (POLAR), insolubility (INSOLU), unsaturation (INSATU), and flexibility (FLEX).

### 2.14. Statistical Analysis

Results were expressed as mean ± standard deviation (SD). Statistical analyses and graphical representations were conducted using GraphPad Prism (version 10, GraphPad Software Inc., Boston, MA, USA). Data distribution normality was verified by Shapiro–Wilk testing. Inter-group comparisons employed one-way analysis of variance with Tukey’s honestly significant difference test for normally distributed data, or the Kruskal–Wallis test with Dunn’s multiple comparisons test for non-normally distributed data. Significance threshold was set at *p* < 0.05. Additionally, Pearson correlation analysis was conducted between allergic asthma and key proteins related to inflammatory and fibrotic signaling pathways using R (version 4.5.1, R Foundation for Statistical Computing, Vienna, Austria), with findings displayed as a correlation heatmap.

## 3. Results

### 3.1. Identification of Bioactive Compounds

**Figure 2:**
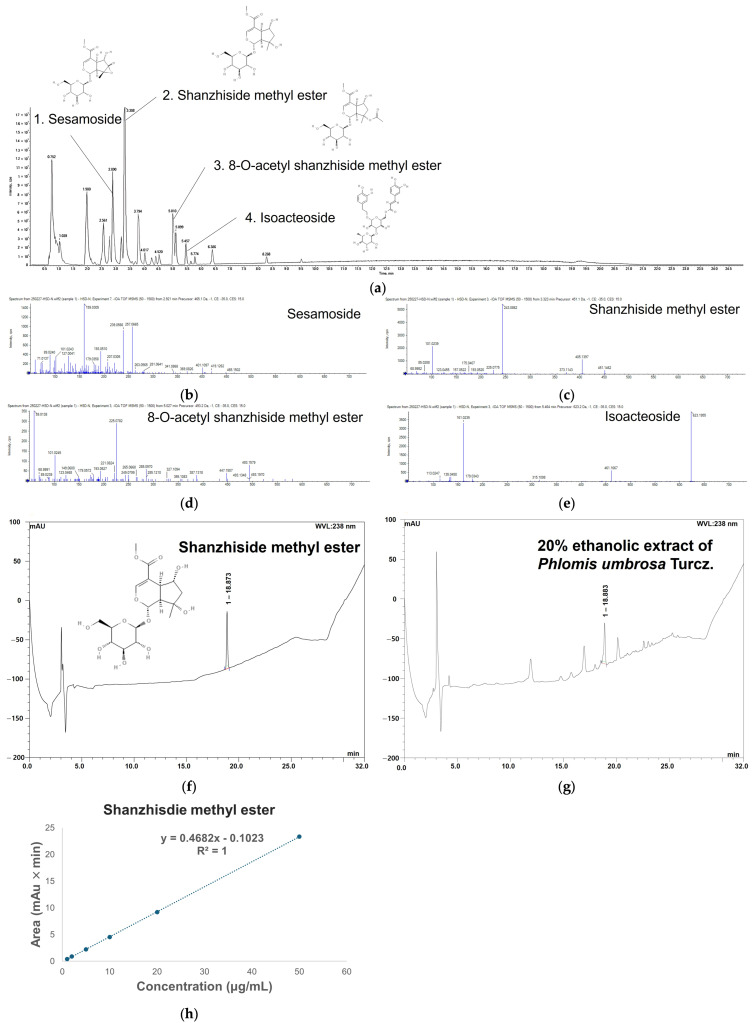
Identification and quantification of bioactive compounds in the 20% ethanolic extract of *Phlomis umbrosa* Turcz. (EPT). (**a**) Ultra performance liquid chromatography-quadrupole time-of-flight tandem-mass spectrometry (UPLC-QTOF-MS/MS) chromatogram displaying major phytochemical components; (**b**–**e**) MS/MS spectra of sesamoside (**b**), shanzhiside methyl ester (**c**), 8-O-acetyl shanzhiside methyl ester (**d**), and isoacteoside (**e**); (**f**,**g**) high-performance liquid chromatography with photodiode array (HPLC-PDA) chromatograms of shanzhiside methyl ester standard (**f**) and EPT (**g**) recorded at 238 nm; (**h**) calibration curve for shanzhiside methyl ester quantitation.

Bioactive compounds of EPT were characterized and identified using UPLC-QTOF-MS/MS, as shown in Figure 2a–e and Table S2. The full, unedited chromatogram is presented in Figure S1. The MS/MS spectra of each compound were analyzed by comparison with the SCIEX OS (Waters Corp.) spectral library and previously reported fragmentation patterns. Phytochemical analysis revealed four major constituents, classified into two structural groups: three iridoid glucosides (sesamoside, shanzhiside methyl ester, and 8-O-acetyl shanzhiside methyl ester) and one phenylethanoid glycoside (isoacteoside). The characteristic negative ion mode MS/MS spectral parameters for these compounds were as follows: sesamoside (RT: 2.890 min; precursor ion: *m*/*z* 465; fragment ions: *m*/*z* 257, 239, 193, and 159), shanzhiside methyl ester (RT: 3.308 min; precursor ion: *m*/*z* 451; fragment ions: *m*/*z* 405, 243, and 101), 8-O-acetyl shanzhiside methyl ester (RT: 5.010 min; precursor ion: *m*/*z* 493; fragment ions: *m*/*z* 225 and 101), and isoacteoside (RT: 5.457 min; precursor ion: *m*/*z* 623; fragment ions: *m*/*z* 461 and 161).

### 3.2. Validation of HPLC Analysis Method

HPLC analysis was performed to quantify shanzhiside methyl ester, the marker compound of *P. umbrosa* in EPT, and was validated. The HPLC-PDA chromatograms and validation parameters are presented in Figure 2f–h and Table 1. A distinct single peak corresponding to shanzhiside methyl ester was clearly observed in both the standard and EPT solution without interference from other constituents, and their RT were nearly similar. Moreover, the ultraviolet spectra of the two samples exhibited a spectral similarity index of 999, thereby confirming the specificity of the analytical method. The correlation coefficient of the calibration line for the standard solution was 1, indicating high linearity. The quantitative analysis was conducted based on a calibration curve, revealing that the shanzhiside methyl ester content in EPT was 5.82 ± 0.10 μg/mg of dry weight. LOD and LOQ were established for sensitivity, and the LOD was worked out to be 0.10 ± 0.00 μg/mL, and the LOQ was worked out to be 0.31 ± 0.00 μg/mL. For precision, repeatability and reproducibility were used as measures. These were calculated to be 0.39% and 0.17%, respectively, suggesting they are within the recommended standards of the AOAC guidelines. Finally, the recoveries at low, medium, and high concentrations were 99.15 ± 1.91%, 99.06 ± 1.53%, and 99.79 ± 2.16%, respectively, indicating that they are within the recovery guidelines established by AOAC [20].

### 3.3. Chemical Structures of Identified Compounds

**Figure 3:**
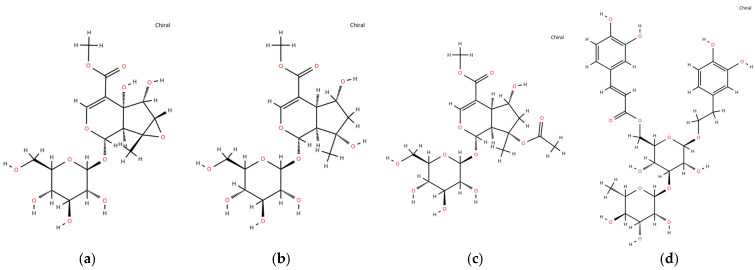
Chemical structures of major phytochemicals identified in EPT. (**a**–**d**) Structures of (**a**) sesamoside, (**b**) shanzhiside methyl ester, (**c**) 8-O-acetyl shanzhiside methyl ester, and (**d**) isoacteoside isolated from EPT.

The chemical structures of the four major phytochemicals identified in EPT are presented in Figure 3 and Table 2. These compounds include three iridoid glucosides (sesamoside, shanzhiside methyl ester, and 8-O-acetyl shanzhiside methyl ester) and one phenylethanoid glycoside (isoacteoside). Structural information, including common names, International Union of Pure and Applied Chemistry (IUPAC) names, molecular formulas, and molecular weights, was compiled from previously reported phytochemical databases and the literature. The IUPAC names of the identified compounds are provided in Table S3.

### 3.4. Effects of EPT Against OVA-Induced Hematological and Biochemical Changes

#### 3.4.1. Alterations of T Lymphocyte Populations

**Figure 4:**
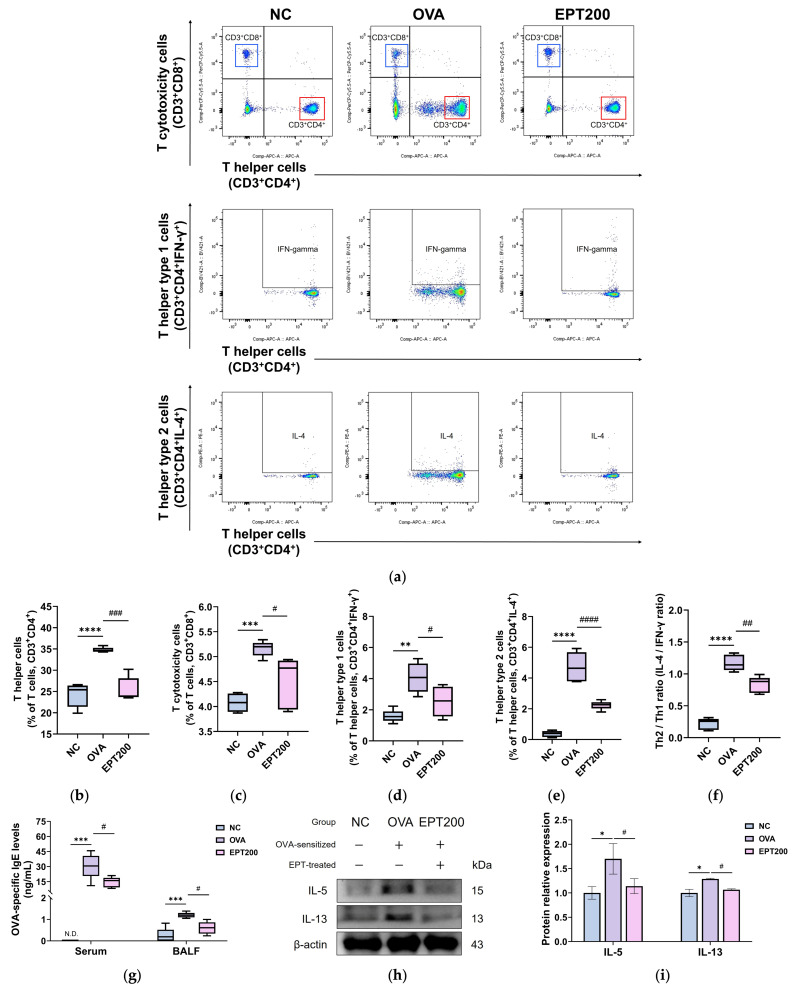
Effect of EPT on T helper type 2 (Th2) immune response due to Th2/T helper type 1 (Th1) imbalance in ovalbumin (OVA)-induced mice. (**a**) Gating strategy for T cell subsets; blue boxes indicate CD3^+^CD8^+^ T cytotoxicity cells, and red boxes indicate CD3^+^CD4^+^ T helper cells; (**b**–**f**) frequency of T helper cells (**b**), T cytotoxicity cells (**c**), Th1 cells (**d**), Th2 cells (**e**), and the Th2/Th1 cells ratio (**f**) in whole blood; (**g**) OVA-specific immunoglobulin E (IgE) levels in serum and bronchoalveolar lavage fluid (BALF); (**h**) representative Western blot images and (**i**) protein expression of Th2-related cytokine, i.e., IL-5 and IL-13, in lung tissues. Values are expressed as mean ± standard deviation (SD) (*n* = 5 for fluorescence-activated cell sorting (FACS) and IgE analysis; *n* = 4 for Western blot analysis). *, **, ***, and **** indicate statistical differences compared with the NC group (*p* < 0.05, 0.01, 0.001, and 0.0001, respectively). #, ##, ###, and #### indicate statistical differences compared with the OVA group (*p* < 0.05, 0.01, 0.001, and 0.0001, respectively). N.D. indicates no detection. EPT200 indicates the OVA-sensitized mice treated with 200 mg/kg EPT.

When an antigen enters the body, T lymphocytes are activated to induce an immune response [4]. Various inflammatory cells are mobilized to the damaged tissues [8]. The subtype ratio of T lymphocytes can be calculated and used to indicate the intensity of the inflammatory response. To evaluate the regulatory effects of EPT on the T helper type 1 (Th1)/Th2 immune balance in OVA-induced allergic asthma mice, the T lymphocyte population in whole blood was assessed. As presented in Figure 4a–f, a significant increase was observed in the percentages of the T cells, including T helper cells, T cytotoxicity cells, Th1 cells, and Th2 cells in the whole blood of the OVA group compared to those of the NC group. Consequently, the Th2/Th1 ratio in the OVA group was markedly elevated compared to the NC group, demonstrating robust Th2 immune responses. However, when EPT was administered, the altered T lymphocyte populations were significantly reduced compared to the OVA group. Also, the Th2/Th1 ratio was significantly compared to the OVA group, indicating that EPT effectively modulated Th2 immune responses in OVA-induced asthmatic mice.

#### 3.4.2. OVA-Specific IgE Levels

IgE is involved in allergic reactions, and elevated OVA-specific IgE levels serve as a parameter of allergen-induced immune activation [7]. To investigate the alleviating effect of EPT on allergic responses, OVA-specific IgE levels were quantified in both serum and BALF of OVA-induced asthmatic mice. As depicted in Figure 4g, the OVA-specific IgE concentration in serum and BALF was markedly increased in the OVA group compared to the NC group. In contrast, EPT administration resulted in a significant reduction in OVA-specific IgE concentration compared to the OVA group, confirming that EPT effectively mitigated OVA-induced allergic reactions.

#### 3.4.3. Th2 Cytokine

Asthma is driven by a Th2-dominant immune response, wherein differentiated Th2 cells secrete IL-5 and IL-13, perpetuating chronic airway inflammation [7]. To assess the regulatory effects of EPT on Th2-mediated cytokine production, the expression levels of Th2 cytokines, specifically IL-5 and IL-13, were analyzed by Western blot analysis. As shown in Figure 4h,I, the expression levels of Th2 cytokines in the lung tissues of the OVA group were markedly elevated compared to the NC group, demonstrating the activation of a Th2-mediated inflammatory response in the lungs. In contrast, EPT administration suppressed the expression levels of these cytokines compared to the OVA group with statistical significance, indicating that EPT effectively suppressed Th2-mediated immune activation in OVA-induced allergic asthma.

#### 3.4.4. WBC Differential Counting

**Figure 5:**
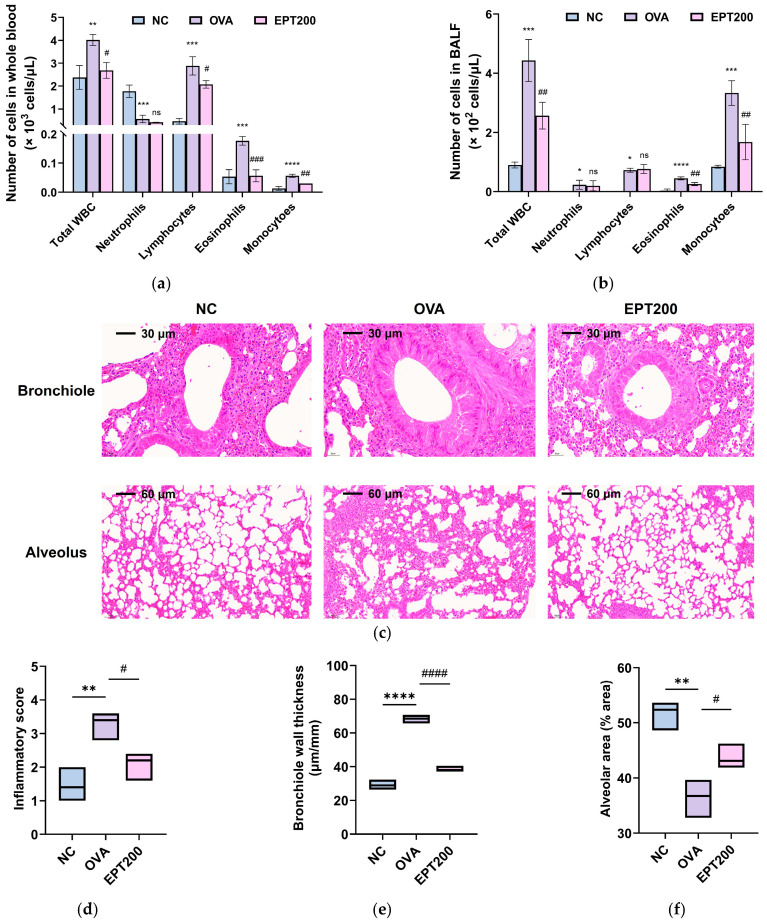
Effect of EPT on histopathological changes due to eosinophilic inflammation in OVA-induced mice. (**a**,**b**) Number of white blood cells (WBCs) in the whole blood (**a**) and BALF (**b**); (**c**) representative hematoxylin and eosin (H&E)-stained images of the bronchiole at 40× magnification (scale bars = 30 μm) and the alveolus at 20× magnification (scale bars = 60 μm); (**d**–**f**) statistical analysis of inflammatory score (**d**), bronchiole wall thickness (**e**), and alveolar area (**f**) in lung tissues. Values are expressed as mean ± SD (*n* = 3 for WBC counts and H&E staining). *, **, ***, and **** indicate statistical differences compared with the NC group (*p* < 0.05, 0.01, 0.001, and 0.0001, respectively). #, ##, ###, and #### indicate statistical differences compared with the OVA group (*p* < 0.05, 0.01, 0.001, and 0.0001, respectively). ns indicates non-significant. EPT200 indicates the OVA-sensitized mice treated with 200 mg/kg EPT.

WBCs are the primary immune cells in the blood, comprising neutrophils, lymphocytes, eosinophils, and monocytes. Changes in the total number of WBCs and their subsets serve as indicators reflecting acute inflammatory responses and immune regulatory status [14]. To evaluate the immunomodulatory effect of EPT in OVA-induced asthmatic mice, the number of WBCs in whole blood and BALF was quantified. As depicted in Figure 5a, the number of total WBC, lymphocytes, eosinophils, and monocytes in the whole blood of the OVA group significantly increased compared to the NC group. On the contrary, EPT administration significantly decreased these cell populations. As shown in Figure 5b, the number of total WBC, eosinophils, and monocytes in the BALF of the OVA group significantly increased compared to the NC group. However, EPT administration significantly decreased these cell populations. There was no significant difference in the number of neutrophils and lymphocytes between the OVA and EPT200 groups.

#### 3.4.5. Histopathological Changes

In patients with asthma, infiltration of inflammatory cells, thickened airway wall, and decreased alveolar area are pathologic markers due to chronic airway inflammation [23]. To examine the protective effects of EPT on histopathological changes, H&E staining was performed and analyzed. As presented in Figure 5c–f, the OVA group exhibited marked histopathological changes, including inflammatory cell infiltration, bronchiole wall thickening, and alveolar septal thickening, suggesting the progressive spread of lung inflammation and fibrosis. When treated with EPT, there was a significant improvement in these histopathological changes compared to the OVA group. Thus, these results indicate that EPT protects against OVA-induced pulmonary structural damage and inflammation in lung tissues.

### 3.5. Effect of EPT Against OVA-Induced Antioxidant System Dysfunction

**Figure 6:**
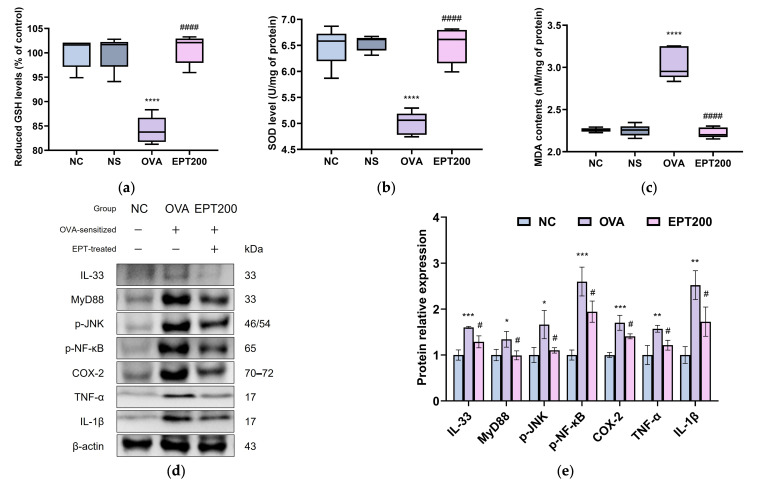
Effect of EPT on oxidative stress and inflammatory response pathway in OVA-induced mice. (**a**–**c**) Antioxidant parameters such as reduced glutathione (GSH) levels (**a**), superoxide dismutase (SOD) levels (**b**), and malondialdehyde (MDA) contents (**c**) in lung tissues; (**d**) representative Western blot images and (**e**) protein expression of inflammation-related pathway, i.e., IL-33, MyD88, p-JNK, p-NF-κB, COX-2, TNF-α, and IL-1β, in lung tissues. Values are expressed as mean ± SD (*n* = 5 for antioxidant parameters; *n* = 4 for Western blot analysis). *, **, ***, and **** indicate statistical differences compared with the NC group (*p* < 0.05, 0.01, 0.001, and 0.0001, respectively). # and #### indicate statistical differences compared with the OVA group (*p* < 0.05, and 0.0001, respectively). EPT200 indicates the OVA-sensitized mice treated with 200 mg/kg EPT.

In allergic asthma, the activation of various inflammatory cells produces excess ROS [1]. This causes oxidative stress, leading to impairment of the antioxidant defense system and contributing to the accumulation of MDA, a marker of oxidative damage [1,11]. To estimate the antioxidant effects of EPT in OVA-induced allergic asthma mice, reduced GSH and SOD levels were determined in lung tissues. As shown in Figure 6a,b, both antioxidant markers in the lung tissue of the OVA group were markedly decreased compared with the NC group, confirming oxidative stress induction. In contrast, EPT administration statistically restored these antioxidant markers. Additionally, MDA contents were measured to assess the degree of oxidative damage. As presented in Figure 6c, the MDA contents in the lung tissue of the OVA group were significantly elevated compared to the NC group. However, EPT administration significantly reduced the MDA content, indicating potent antioxidant activity. Collectively, this indicates that EPT effectively mitigates oxidative stress induced lung injury by enhancing antioxidant enzyme activity and reducing lipid peroxidation.

### 3.6. Effect of EPT Against OVA-Induced Pulmonary Inflammation-Related Factors

Exposure to allergens and oxidative stress can damage airway epithelial cells, triggering the secretion of IL-33, which subsequently activates the NF-κB signaling pathway [24]. This cascade promotes the transcription of downstream inflammatory mediators, thereby contributing to the pathogenesis of asthma [5]. To elucidate the molecular mechanisms underlying the anti-inflammatory effects of EPT in OVA-induced allergic asthma mice, the expression of proteins involved in the IL-33/NF-κB signaling pathway was examined by Western blot analysis. As depicted in Figure 6d,e, the expression levels of IL-33, myeloid differentiation primary response 88 (MyD88), p-JNK, p-NF-κB, cyclooxygenase-2 (COX-2), tumor necrosis factor alpha (TNF)-α, and IL-1β in the lung tissues of the OVA group were markedly upregulated compared to the NC group, indicating activation of pulmonary inflammatory signaling. In contrast, EPT administration statistically significantly downregulated the expression of these proteins compared to the OVA group. This demonstrates that EPT effectively attenuates airway inflammation in OVA-induced asthmatic mice by suppressing the IL-33/NF-κB pathway.

### 3.7. Effect of EPT Against OVA-Induced Pulmonary Fibrosis-Related Factors

**Figure 7:**
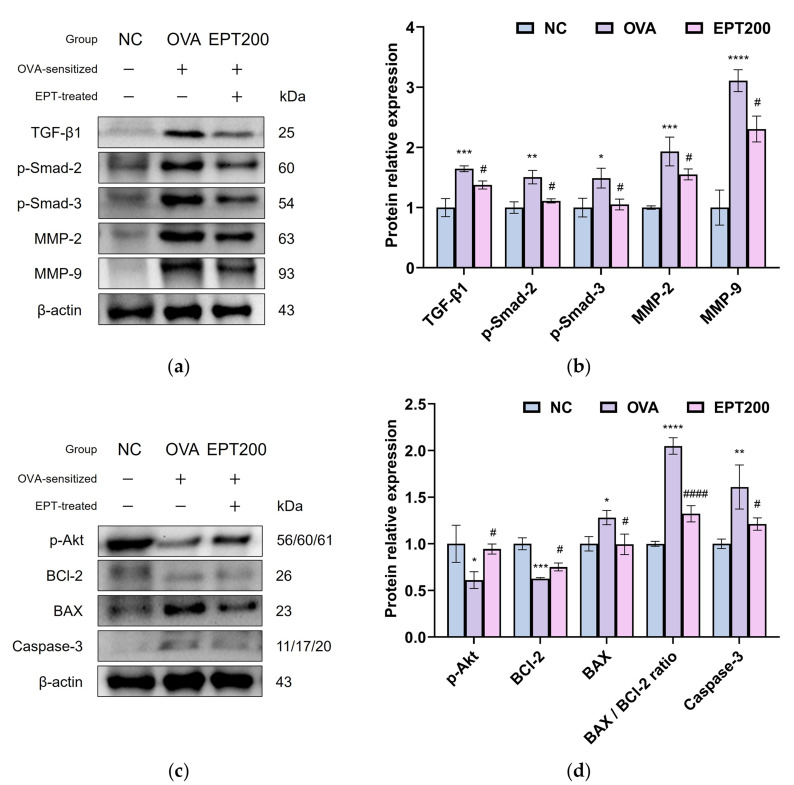
Effect of EPT on pulmonary fibrosis and apoptosis pathway in OVA-induced mice. (**a**) Representative Western blot images of fibrosis and (**b**) protein expression of pulmonary fibrosis-related pathway, i.e., TGF-β1, p-Smad-2, p-Smad-3, MMP-2, and MMP-9, in lung tissues; (**c**) representative Western blot images of apoptosis and (**d**) protein expression of apoptosis-related pathway, i.e., p-Akt, BC1-2, BAX, BAX/BCI-2 ratio, and caspase-3, in lung tissues. Values are expressed as mean ± SD (*n* = 4). *, **, ***, and **** indicate statistical differences compared with the NC group (*p* < 0.05, 0.01, 0.001, and 0.0001, respectively). #, and #### indicate statistical differences compared with the OVA group (*p* < 0.05, and 0.0001, respectively). EPT200 indicates the OVA-sensitized mice treated with 200 mg/kg EPT.

Chronic inflammation and repetitive apoptosis lead to lung tissue destruction and fibrosis, and the TGF-β1/Smad signaling pathway is used as an indicator of the fibrotic mechanism [25]. To demonstrate the molecular mechanisms underlying the anti-fibrotic effects of EPT in OVA-induced allergic asthma mice, the expression of proteins related to the TGF-β1/Smad signaling pathway was analyzed by Western blot analysis. As shown in Figure 7a,b, the expression levels of TGF-β1, p-Smad-2, p-Smad-3, matrix metalloproteinase (MMP)-2, and MMP-9 in the lung tissues of the OVA group were increased compared to the NC group, indicating the progression of pulmonary fibrosis. In contrast, EPT administration exhibited statistically meaningful inhibition of the expression of these proteins compared to the OVA group. These results indicate that EPT effectively impedes airway fibrosis in OVA-induced allergic asthma mice by regulating the TGF-β1/Smad signaling pathway.

### 3.8. Effect of EPT Against OVA-Induced Pulmonary Apoptosis-Related Factors

Increased levels of Th2 cytokines are responsible for mitochondrial defects, which promote the apoptosis of airway cells and contribute to the pathogenesis of asthma and lung remodeling [26]. To clarify the molecular mechanisms underlying the anti-apoptotic effects of EPT in OVA-induced allergic asthma, the expression of proteins related to apoptosis was investigated by Western blot analysis. As presented in Figure 7c,d, the expression levels of p-protein kinase B (p-Akt) and B-cell leukemia/lymphoma 2 (BCl-2) in the lung tissues of the OVA group were decreased compared to the NC group, while increasing BCl-2 associated X (BAX) and caspase-3 compared to the NC group. The BAX/BCl-2 ratio in the lung tissues of the OVA group was markedly elevated compared to the NC group, indicating enhanced apoptotic signaling in the lungs. Conversely, EPT treatment significantly reversed the expression of these proteins compared to the OVA group, thereby reducing the BAX/BCl-2 ratio relative to the OVA group. These results demonstrate that EPT effectively mitigates apoptosis in OVA-induced allergic asthma mice by modulating the BAX/BCl-2 ratio.

### 3.9. Pearson Correlation Analysis Between Allergic Asthma and Key Signaling Pathways

**Figure 8:**
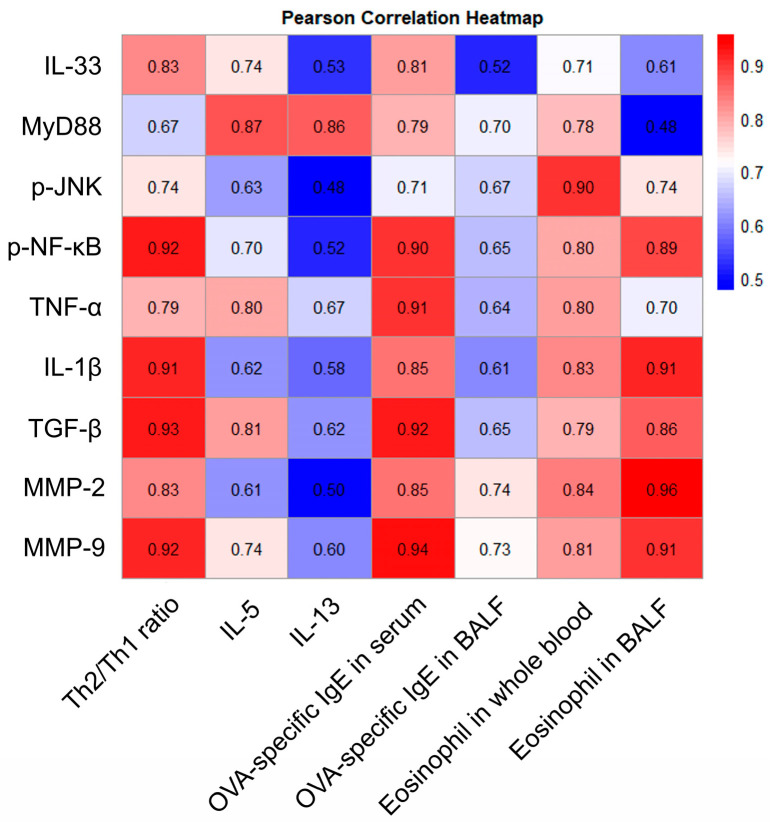
Pearson correlation analysis between allergic asthma and representative markers of inflammation and fibrosis.

Based on the abovementioned information, a Pearson correlation analysis was performed to investigate changes induced by OVA exposure. This analysis aimed to better understand the relationship between allergic asthma parameters and inflammation and fibrosis pathways. As shown in Figure 8, positive correlations were observed among immune, oxidative stress, inflammatory, and fibrotic parameters, with correlation coefficients exceeding 0.5. Indicators of allergic asthma, which are the Th2/Th1 ratio, levels of OVA-specific IgE, and numbers of eosinophils, exhibited significant positive correlations with inflammation and fibrosis. These results suggest that the Th2-biased immune response plays a central role in the pathological progression of allergic asthma by amplifying inflammatory and fibrotic responses.

### 3.10. SwissADME Studies of the Isolated Compounds

**Figure 9:**
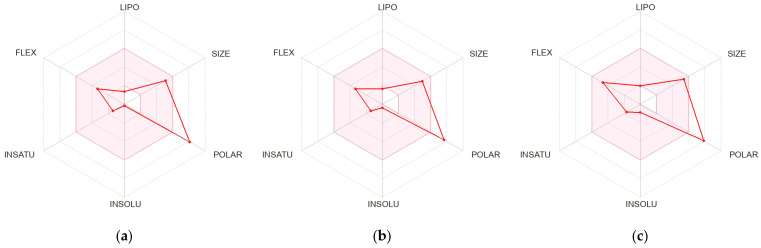
Physicochemical properties radar chart of isolated compounds. (**a**) Sesamoside; (**b**) shanzhiside methyl ester; (**c**) 8-O-acetyl shanzhiside methyl ester.

To further characterize bioactive compounds and assess their suitability as functional material candidates, their physicochemical and pharmacokinetic profiles were evaluated using SwissADME. Table 3 summarizes the predicted ADME-related physicochemical and pharmacokinetic properties of the three major phytochemicals identified in EPT, namely sesamoside, shanzhiside methyl ester, and 8-O-acetyl shanzhiside methyl ester, and their physicochemical characteristics are illustrated in Figure 9. The molar refractivity values ranged from 87.57 for sesamoside to 98.30 for 8-O-acetyl shanzhiside methyl ester. All three compounds exhibited high TPSA values of 187.90 Å^2^ for sesamoside, 175.37 Å^2^ for shanzhiside methyl ester, and 181.44 Å^2^ for 8-O-acetyl shanzhiside methyl ester, and consensus Log P*_o_*_/_*_w_* values ranged from −2.28 to −1.39, reflecting their hydrophilic nature. Accordingly, all compounds were predicted to have low GI absorption, with identical bioavailability scores of 0.11, and none were predicted to penetrate the BBB, as further corroborated by the radar charts in Figure 9, which show extended profiles in the POLAR and INSOLU axes and compressed profiles in the LIPO dimension. Regarding metabolic interactions, none of the compounds were predicted to inhibit CYP1A2, CYP2C19, CYP2C9, CYP2D6, or CYP3A4, and none were identified as P-gp substrates. PAINS analysis revealed no structural alerts for any of the three compounds, while Brenk alerts were detected in sesamoside and 8-O-acetyl shanzhiside methyl ester but not in shanzhiside methyl ester. Overall, these results suggest that although the three compounds exhibit low predicted oral absorption, they exhibit favorable profiles for metabolic enzyme inhibition, efflux transport, and assay interference, supporting their potential as bioactive natural product leads.

## 4. Discussion

Irrespective of age, asthma is a chronic respiratory disease that affects a significant proportion of the population [2]. Asthma has a highly diverse pathogenesis [10]. Allergic asthma, which develops in response to allergens, is characterized by the activation and infiltration of immune cells, as well as chronic airway inflammation [3]. In the case of prolonged inflammatory conditions, the massive infiltration of inflammatory cells produces ROS that induces oxidative stress [26]. Oxidative stress accelerates the immune response, contributing to pulmonary fibrosis and apoptosis, which in turn result in airway remodeling and decreased lung function [3,12]. Bioactive phytochemicals from plants exhibit anti-inflammatory, antioxidant, and other biological activities by modulating signaling pathways associated with multiple molecular targets [27]. Therefore, botanical extracts have gained attention as therapeutic alternatives for asthma with fewer side effects compared to pharmaceutical drugs [10]. Accordingly, our study established an OVA-induced allergic asthma model to evaluate the protective potential of EPT.

*P. umbrosa*, has various biological properties, such as anti-allergic, anti-inflammatory, and detoxification properties [28]. These effects are attributed to bioactive substances contained in the roots of *P. umbrosa* [19]. In our study, we used the UPLC-QTOF-MS/MS analysis to identify the bioactive compounds in EPT. We identified four bioactive compounds, sesamoside, shanzhiside methyl ester, 8-O-acetyl shanzhiside methyl ester, and isoacteoside (Figure 2a–e and Figure 3, and Table 2). Isoacteoside is a phenylethanoid glycoside with a glycosyl group bound to a phenylethyl alcohol skeleton [29]. The phenolic hydroxyl group in its structure exhibits antioxidant activity by scavenging free radicals [30]. Other bioactive compounds, sesamoside, shanzhiside methyl ester, and 8-O-acetyl shanzhiside methyl ester, are iridoid glucosides characterized by a cyclopentane pyran ring structure [31]. The bioactive properties of shanzhiside, including anti-inflammatory and immunomodulatory effects, are linked to the C8-positioned hydroxyl functionality in its glucoside structure [32]. Its methylated derivative, shanzhiside methyl ester, is known to be a marker compound of *P. umbrosa* [18]. We developed an HPLC-PDA method for this compound and validated it according to the AOAC guidelines (Figure 2f–h and Table 1). Collectively, these parameters confirm the robustness and reliability of the analytical method. Previous studies reported that shanzhiside methyl ester inhibits inflammatory mediators in neutrophils and exerts anti-inflammatory effects [31,33]. Additionally, Pak et al. [17] previously reported the anti-asthmatic effects of *P. umbrosa* extract using an OVA-induced mouse model. However, the extraction conditions used in this study differ from those reported previously. While Pak et al. [17] employed a 70% ethanol extract of *P. umbrosa*, we utilized a 20% ethanol extract. This extraction strategy was selected with consideration of potential industrial applicability, as lower ethanol concentrations may offer advantages for large-scale production by reducing solvent costs and improving process safety during manufacturing. Moreover, in contrast to previous studies that performed phytochemical analyses without full analytical validation, this study established and validated an HPLC method for the quantitative analysis of the major bioactive compound, shanzhiside methyl ester, in accordance with AOAC guidelines. This validation improves the reliability and reproducibility of the phytochemical characterization of EPT. Therefore, the extraction approach used in this study may provide additional practical value for the development of functional materials derived from *P. umbrosa*. Collectively, these methodological differences extend the findings of the previous study by providing improved analytical reliability and practical considerations for the potential industrial utilization of *P. umbrosa* extracts. Based on these considerations, we aimed to investigate whether EPT containing shanzhiside methyl ester improves lung function by modulating the Th2 immune response in an OVA-induced allergic asthma mouse model.

OVA-sensitized mice closely replicate the pathological characteristics of human asthma, making them a widely used model for investigating disease pathogenesis and evaluating therapeutic interventions [6,8]. Upon allergen exposure, damaged airway epithelial cells release alarmins such as IL-33, which activate DCs, Th2, and type 2 innate lymphoid cells [34]. Activated DCs subsequently migrate to draining lymph nodes [4]. Naïve CD4^+^ T cells differentiate into Th2 cells through T cell receptor signaling, co-stimulatory signals from DCs, and IL-4 from follicular T helper cells, while Th1 polarization is simultaneously suppressed [7]. Recent studies reported that during acute activation, T cytotoxicity cells transiently respond to IL-33 and enhance type 2 cytokine production [34]. This cascade of events disrupts the Th1/Th2 balance, amplifying the Th2-dominant immune response [6]. The resulting imbalance drives the production of OVA-specific IgE and Th2 cytokines, including IL-4, IL-5, and IL-13, which are critical mediators in the pathophysiology of asthma [24]. Consistent with this mechanism, our study found that OVA exposure induced the activation of T lymphocytes and differentiation of Th2 cells, leading to a characteristic Th2 immune response (Figure 4). Elevated OVA-specific IgE levels and increased expression of Th2-associated cytokines evidenced this. In contrast, EPT treatment effectively suppressed these Th2 immune responses. Our study is consistent with reports that the water extract of *P. umbrosa* suppresses Th2 cytokine expression in RAW 264.7 macrophages stimulated with lipopolysaccharide (LPS) and in a murine model of OVA-induced hypersensitivity [35]. Similarly, Shin et al. [36] reported that when a systemic allergic response is induced by compound 48/80, a degranulating agent of mast cells, an aqueous extract of *P. umbrosa* alleviates the response by blocking the release of histamine and inflammatory cytokines from activated mast cells. This suggests that EPT could alleviate Th2 bias, modulate the differentiation of Th cells, and block a key pathological mechanism of asthma.

Inflammation plays a pivotal role in the development and progression of allergic asthma [10]. IL-5, a key Th2 cytokine, drives eosinophil recruitment from the bloodstream into the airways, leading to eosinophilia [37]. Infiltrated eosinophils secrete various mediators to induce eosinophilic inflammation and contribute to histopathological changes in asthma [38]. Notably, this eosinophilic inflammation affects not only the central airways but also extends distally to the bronchioles and alveolar parenchyma [39]. Consistent with these reports, we observed that OVA sensitization was accompanied by eosinophilia, inflammatory cell infiltration, and a reduction in alveolar space size [40]. These changes are likely attributed to the accumulation of the extracellular matrix (ECM) [40]. Eosinophils undergo degranulation and release eosinophil cationic granule proteins, which stimulate mast cells to secrete histamine and amplify type 2 inflammatory response [23]. Eosinophils also secrete TGF-β1 and MMPs, which promote ECM accumulation and contribute to airway remodeling [38]. In our study, OVA exposure increased eosinophil counts and altered the structure of bronchioles (Figure 5). This finding underscores the pivotal role of eosinophilic inflammation in the development of asthma. We observed that treatment with EPT alleviated these biochemical and histopathological changes. This aligns with the observations by Pak et al. [17], which demonstrated that a 70% ethanolic extract of *P. umbrosa* effectively suppressed eosinophilia and inflammatory cytokine production, alleviating pulmonary inflammation and mucus secretion. Furthermore, the Lamiaceae family, which includes *P. umbrosa*, is shown to have potential therapeutic effects on allergic diseases by inhibiting eosinophil infiltration and reducing mucus hypersecretion, goblet cell proliferation, and bronchial hyperreactivity in lung tissue [41]. The combined results of our studies and those of previous studies suggest that EPT can effectively mitigate tissue damage associated with the Th2 immune response in allergic asthma.

Oxidative stress is closely associated with allergic inflammation [11]. Upon allergen exposure, inflammatory cells generate excess ROS, disrupting the redox balance and inducing oxidative stress [1]. This further amplifies inflammation by upregulating IL-33 expression, which is involved in Th2 immune signaling [42,43]. IL-33 binds to the IL-1 receptor-like 1 and activates JNK and NF-κB through a MyD88-dependent pathway [43]. This pathway promotes the secretion of not only Th2 cytokines but also inflammatory cytokines, including COX-2, TNF-α, and IL-1β, to induce chronic inflammation [5,43]. Meanwhile, the body has antioxidant defense systems such as GSH and SOD to mitigate the damage induced by oxidative stress [1]. Antioxidant defense systems are impaired in asthma patients, which leads to elevated MDA levels, a biomarker of oxidative stress [11]. Oxidative stress interacts with inflammatory responses to form a pathological vicious cycle, which exacerbates allergic asthma [11]. Consistent with these mechanisms, we observed that OVA exposure activated the IL-33-mediated inflammatory pathway, weakened the antioxidant defense system, and increased MDA levels (Figure 6). However, treatment with EPT suppressed the inflammatory pathway and restored the impaired antioxidant system. As mentioned earlier, this could probably be due to the bioactive compounds of *P. umbrosa*. Isoacteoside exhibits antioxidant activity by scavenging free radicals through phenolic hydroxyl groups in its structure [29,30]. Similarly, isoacteoside alleviated lung injury and pulmonary edema by increasing endogenous antioxidants such as catalase (CAT), SOD, and GSH and inhibiting MDA in LPS-challenged acute lung injury mice [44]. Indeed, the water extract of *P. umbrosa* enhanced the activities of antioxidant enzymes, including SOD, CAT, and glutathione peroxidase, and suppressed inflammatory pathway proteins in OVA-sensitized hypersensitivity mice [35]. In addition, unlike the previous study, which mainly proposed potential mechanisms of *P. umbrosa* via network pharmacology analysis [17], this study experimentally investigated several key molecular mechanisms associated with allergic asthma in an in vivo model. Specifically, we verified the involvement of the IL-33/MyD88/NF-κB axis associated with epithelial-derived alarmin signaling, providing experimental evidence for the regulation of this pathway by EPT. Therefore, EPT appears to alleviate lung damage caused by allergic inflammation by suppressing oxidative stress and inflammatory responses.

Chronic airway remodeling is a hallmark histopathological feature of allergic asthma, characterized by structural alterations that include smooth muscle hypertrophy, epithelial injury, and subepithelial fibrosis [45]. TGF-β1 is a key mediator of subepithelial fibrosis [46]. Inflammation converts TGF-β1 into its active form, which accumulates in asthmatic airways [47]. Its expression levels correlate with basement membrane thickness and disease severity [48]. TGF-β activates the canonical Smad2/3 pathway, promoting epithelial–mesenchymal transition and ECM accumulation in airway walls [25]. Concurrently, TGF-β stimulates alveolar macrophages to produce MMP-2 and MMP-9, thereby enhancing ECM degradation and further contributing further to structural remodeling [12]. The resulting pulmonary fibrosis impairs lung parenchymal architecture, manifesting as respiratory dysfunction [45,48]. In agreement with this, OVA exposure elevated the expression of TGF-β/Smad pathway-associated proteins (Figure 7a,b). This suggests that inflammation caused by the Th2 immune responses exacerbated fibrosis. However, treatment with EPT suppressed fibrosis progression by attenuating TGF-β/Smad signaling. These effects are presumed to be a result of the diverse bioactive compounds in *P. umbrosa*. In related research, Chen et al. [30] reported that isoacteoside exerts anti-fibrotic effects by regulating the expression of type I collagen through the inhibition of the Smad and non-Smad pathways in mouse and human lung fibroblasts. Furthermore, shanzhiside methyl ester and 8-O-acetyl shanzhiside methyl ester suppressed CCl_4_-induced hepatic fibrosis by regulating ECM-associated factors, including laminin, collagen IV, and smooth muscle proteins [49]. Interestingly, Pak et al. [17] reported that *P. umbrosa* extract reduced pulmonary inflammation, mucus secretion, and MMP-9 expression in OVA-induced asthmatic mice. However, the molecular mechanisms underlying these observations remain unclear. In this study, we demonstrated that EPT suppresses TGF-β/Smad signaling associated with pulmonary fibrosis, providing mechanistic insight that may partially explain the previously reported reduction in MMP-9 expression and airway remodeling. Taken together, our findings demonstrate that EPT exerts anti-fibrotic effects in allergic asthma by targeting TGF-β/Smad-mediated pulmonary fibrosis.

Beyond the canonical Smad pathway, TGF-β also modulates cell survival and apoptosis via non-Smad signaling, particularly through the phosphoinositide 3-kinase (PI3K)/Akt pathway [47]. Although the PI3K/Akt pathway promotes cell survival in normal tissue regeneration, it is reportedly inhibited in inflammatory environments, such as the airways of patients with asthma, leading to apoptosis induction [47,50]. This phenomenon was evident in OVA-induced chronic asthma, where Akt phosphorylation was diminished [51]. Concurrently, the balance between apoptotic regulators shifted, with BCl-2 downregulation and BAX upregulation characterizing the pathology of allergic asthma [51]. These molecular changes culminate in mitochondrial caspase-3 activation, propagating apoptosis [26]. Ultimately, intensifying pulmonary inflammation and fibrosis deteriorates lung function [26]. Consistent with these apoptotic mechanisms, OVA exposure increased the expression of apoptosis-related proteins (Figure 7c,d). In contrast, EPT treatment downregulated the apoptotic pathway and alleviated OVA-induced lung cell damage. In this regard, isoacteoside mitigated against glutamate-induced apoptosis in PC12 cells by modulating the BCl-2/BAX ratio and cleaved caspase-3 levels [52]. Similarly, 8-O-acetyl shanzhiside methyl ester reduced apoptosis and restored mitochondrial bioenergetics in oxygen and glucose-deprived rat cortical neurons through caspase-3 inhibition [53]. These findings support the potential role of bioactive constituents of *P. umbrosa* in regulating apoptosis-related signaling pathways. In this context, this study extends mechanistic insights into apoptosis-related signaling pathways previously predicted through network pharmacology analysis by Pak et al. [17]. In addition to fibrosis-associated signaling, we investigated the Akt/BAX/Bcl-2 axis and demonstrated that EPT attenuates apoptosis in lung tissues during allergic inflammation. Therefore, EPT may serve as a promising natural therapeutic candidate that attenuates apoptosis through the anti-inflammatory and antifibrotic properties of its bioactive constituents, thereby preventing lung damage in allergic asthma.

Finally, to further broaden the mechanistic interpretation of the findings, correlation network analysis was performed between allergic asthma markers and inflammation- and fibrosis-related parameters. Pearson correlation analysis revealed that allergic asthma is significantly correlated with inflammation and fibrosis (Figure 8). In particular, p-NF-κB showed the strongest correlation with parameters of allergic asthma, exhibiting high positive correlations with the Th2/Th1 ratio, OVA-specific IgE in serum, and number of eosinophils in BALF. Additionally, TGF-β and MMP-9 exhibited strong positive correlations with these parameters. These correlation patterns underscore the central roles of NF-κB-mediated inflammation and TGF-β/MMP-driven fibrosis in asthma pathogenesis [45,54]. Collectively, this correlation network demonstrates that Th2 immune responses, inflammation, and fibrosis operate as an interconnected pathological axis in allergic asthma. This approach provides a more integrated understanding of the inflammation-fibrosis axis in allergic asthma by identifying significant associations among immune, inflammatory, and fibrotic indicators. Our findings demonstrate that EPT ameliorates allergic asthma through dual mechanisms: suppression of Th2-driven inflammation and inhibition of TGF-β/Smad-mediated fibrosis. These findings, combined with the identification of bioactive constituents such as shanzhiside methyl ester and isoacteoside, establish the potential of EPT as a plant-derived candidate for the management of allergic asthma.

Based on these results, pharmacodynamic analyses were performed to evaluate the potential of the identified compounds as functional material candidates. ADME analysis supports drug candidate selection by predicting key pharmacokinetic characteristics [55]. Compounds that satisfy classical rules, which are Lipinski’s rule of five, Ghose filter, Veber’s rule, and Muegge’s rule, are generally considered to have favorable pharmacokinetic potential for oral administration [55]. However, this rule was primarily developed for small synthetic molecules and has limited applicability to natural product-derived compounds with higher molecular weights and more complex structures [56]. Consequently, many plant-derived compounds fail to meet these rules, as observed for the iridoid glycosides identified in this study (Table 3 and Figure 9) [57]. Natural products often exhibit high TPSA and low lipophilicity, which may reduce membrane permeability and lead to lower predicted GI absorption and bioavailability [58]. Consistent with these characteristics, the three compounds analyzed in this study showed high TPSA values and low Log P*_o_*_/_*_w_* values, resulting in low predicted GI absorption. Nevertheless, significant biological activity was observed in vivo, likely attributable to gut microbial β-glucosidase-mediated deglycosylation of iridoid glycosides into bioactive aglycone forms [31]. Thus, low predicted absorption reflects metabolism-dependent activation rather than reduced efficacy. In addition, the low lipophilicity of these compounds was predicted to limit BBB permeability, thereby restricting neurological applications but potentially reducing central nervous system-related adverse effects and favoring peripheral anti-inflammatory activity [59]. From a metabolic perspective, CYP450 enzymes play a crucial role in the hepatic biotransformation of xenobiotics [60]. In this study, the three analyzed compounds were predicted not to inhibit major CYP450 isoforms, including CYP1A2, CYP2C9, CYP2C19, CYP2D6, and CYP3A4, suggesting a minimal risk of metabolic interference or drug–drug interactions [61]. Furthermore, P-gp functions as a key efflux transporter that pumps drugs back into the intestinal lumen, thereby reducing their plasma and tissue concentrations [62]. The prediction that these compounds are not P-gp substrates suggests that efflux transport does not limit their pharmacokinetic properties [56]. These findings suggest a low risk of adverse effects when co-administered with other drugs and indicate that the compounds may serve as safe, natural product-derived candidates with minimal potential for in vivo toxicity. Importantly, the PAINS analysis showed “0 alert” for all three compounds, indicating the absence of problematic substructures that could cause nonspecific interactions or false-positive results in biological assays [55]. This suggests that the observed anti-asthmatic effects likely stem from genuine interactions with biological targets. In addition, the Brenk filter identifies structural fragments potentially associated with toxicity, chemical reactivity, or metabolic instability by screening compounds against 105 structural alerts proposed by Brenk et al. [62,63]. Although a single Brenk alert was identified for sesamoside due to its epoxide group and for 8-O-acetyl-shanzhiside methyl ester owing to its multiple ester moieties, these findings likely reflect the structural complexity inherent to bioactive iridoids [32]. Despite predicted pharmacokinetic limitations, EPT demonstrated meaningful in vivo efficacy in the OVA-induced allergic asthma model, accompanied by modulation of key mechanistic pathways. These findings suggest that the identified compounds achieve sufficient biological activity in vivo despite their relatively low predicted oral absorption. While the compounds show potential as respiratory anti-inflammatory candidates, further empirical validation of their metabolic stability and pharmacokinetic behavior is warranted, as the relationship between their pharmacokinetic properties and in vivo mechanistic effects remains to be fully elucidated. In particular, considering that many plant-derived glycosides can be metabolized by the gut microbiota into bioactive metabolites, future studies should investigate the potential interactions between these compounds and intestinal microbial metabolism. Furthermore, strategies to improve bioavailability, such as structural modification, formulation optimization, or delivery system development, may enhance their therapeutic potential.

Nevertheless, several limitations of this study should be acknowledged. First, a standard pharmacological comparator, such as dexamethasone, was not included as a positive control, limiting the ability to directly compare the efficacy of EPT with established anti-inflammatory agents. This limitation may limit the clinical applicability of the present findings. Second, although phosphorylation levels of key signaling proteins were analyzed, the corresponding total protein levels were not measured, and therefore, phospho/total protein ratios could not be calculated. Third, the sample sizes used in certain analyses were relatively small due to methodological constraints, potentially limiting the statistical power of the findings. Finally, formal taxonomic authentication by a qualified botanist and deposition of a voucher specimen in a recognized herbarium were not performed for the plant material used in this study, which may limit the taxonomic documentation and reproducibility of the findings. In addition, as this study was conducted in experimental mouse models, the lack of human validation or clinical correlation remains a limitation. From a translational perspective, EPT may be applicable both as a functional food ingredient and as a standardized therapeutic extract, given its plant-derived origin and demonstrated anti-inflammatory properties. However, further studies are needed for clinical translation. The predicted low oral bioavailability of the major bioactive compounds underscores the need for formulation strategies, such as encapsulation or nanoparticle-based delivery, to enhance absorption. Additionally, optimal dosing regimens have yet to be established, as the relationship between administered dose and systemic exposure remains unclear in humans. Addressing these challenges will be essential for advancing EPT-based candidates toward clinical application.

Collectively, this study suggests that EPT exerts anti-asthmatic effects by modulating multiple signaling pathways. Specifically, EPT suppresses IL-33-mediated NF-κB signaling, thereby attenuating inflammatory responses, while simultaneously inhibiting TGF-β/Smad signaling to reduce airway remodeling and fibrosis. In addition, EPT regulates apoptosis-related pathways by modulating the Akt/BAX/BCl-2 axis, thereby contributing to the protection of airway structural integrity. These integrated mechanisms are also summarized in the graphical abstract. Overall, this study suggests that EPT may be a promising plant-derived functional material for the management of allergic asthma.

## 5. Conclusions

Our study demonstrates that EPT alleviates OVA-induced allergic asthma. The Th2 immune response, characteristic of allergic asthma, was effectively controlled by EPT through suppression of OVA-triggered T lymphocyte activation. EPT significantly ameliorated airway inflammation and structural alterations by limiting the mobilization and activation of diverse inflammatory cell populations. Furthermore, EPT restored the compromised antioxidant defense mechanisms in pulmonary tissues affected by the activation of inflammatory cells and the production of ROS by the Th2 inflammatory response. Molecularly, EPT attenuated Th2-mediated inflammatory cascades via downregulation of IL-33-dependent JNK and NF-κB signaling. Additionally, EPT modulated the TGF-β1/Smad signaling pathway and the ratio of BAX/BCl-2 to mitigate fibrosis and apoptosis in lung tissues. These protective properties of EPT stem from its bioactive constituents, including sesamoside, shanzhiside methyl ester, 8-O-acetyl shanzhiside methyl ester, and isoacteoside. Collectively, our study suggests that EPT exerts anti-asthmatic properties by alleviating inflammation, fibrosis, and apoptosis. The results demonstrate that EPT is a promising natural plant material that can be used as a functional food ingredient to manage allergic asthma.

## Figures and Tables

**Table 1:** Validation parameters of HPLC analysis for shanzhiside methyl ester.

Parameters	Shanzhiside Methyl Ester		
Linearity range (μg/mL)	1–50		
Regression equation	y = 0.4682x – 0.1023		
Contents (μg/mg of dry weight)	5.82 ± 0.10		
Correlation coefficient (R^2^)	1		
Repeatability (%)	0.39		
Reproducibility (%)	0.17		
Limit of detection (μg/mL)	0.10 ± 0.00		
Limit of quantification (μg/mL)	0.31 ± 0.00		
Recovery rate (%)	Concentration (μg/mL)		
	27.2	34	40.8
	99.15 ± 1.91	99.06 ± 1.53	99.79 ± 2.16

Values are expressed as mean ± SD (*n* = 3).

**Table 2:** Chemical information of major phytochemicals identified in EPT.

Compound	Molecular Formula	Molecular Weight
Sesamoside	C_17_H_24_O_12_	420.4 g/mol
Shanzhisid methyl ester	C_17_H_26_O_11_	406.4 g/mol
8-O-acetyl shanzhiside methyl ester	C_19_H_28_O_12_	448.4 g/mol
Isoacteoside	C_29_H_36_O_15_	624.6 g/mol

**Table 3:** The absorption, permeability, and enzyme interaction profiles of phytochemical compounds of EPT.

	Sesamoside	Shanzhiside Methyl Ester	8-O-Acetyl Shanzhiside Methyl Ester
Molar refractivity	87.57	88.57	98.30
TPSA	187.90	175.37	181.44
Consensus Log P*_o_*_/_*_w_*	−2.28	−1.72	−1.39
GI absorption	Low	Low	Low
Bioavailability score	0.11	0.11	0.11
BBB permeant	No	No	No
CYP1A2 inhibitor	No	No	No
CYP2C19 inhibitor	No	No	No
CYP2C9 inhibitor	No	No	No
CYP2D6 inhibitor	No	No	No
CYP3A4 inhibitor	No	No	No
P-gp substrate	No	No	No
PAINS	0 alert	0 alert	0 alert
Brenk	1 alert; Three-membered_heterocycle	0 alert	1 alert; more_than_2_esters

## Data Availability

The original contributions presented in this study are included in the article and Supplementary Materials. Further inquiries can be directed to the corresponding author.

## Supplementary Materials

The following supporting information can be downloaded at: https://www.mdpi.com/article/10.3390/antiox15040420/s1. Figure S1: The ultra-performance liquid chromatography–quadrupole time-of-flight tandem–mass spectrometry (UPLC-QTOF-MS/MS) chromatograms of the 20% ethanolic extract of *Phlomis umbrosa* Turcz. (EPT) (a). (b–e) Fragment ion peaks from sesamoside (b), shanzhiside methyl ester (c), 8-O-acetyl shanzhiside methyl ester (d), and isoacteoside (e); Table S1: Details of antibodies utilized in this study; Table S2: Identification of main compounds in the 20% ethanolic extract of *Phlomis umbrosa* Turcz. (EPT) using ultra-performance liquid chromatography–quadrupole time-of-flight tandem–mass spectrometry (UPLC-QTOF-MS/MS) analysis; Table S3. International Union of Pure and Applied Chemistry (IUPAC) names of the major phytochemicals identified in the 20% ethanolic extract of *Phlomis umbrosa* Turcz. (EPT).

## Abbreviations

Akt	Protein kinase B
AOAC	Association of Official Analytical Chemists
BCl-2	B-cell leukemia/lymphoma 2
BAX	BCl-2 associated X
BALF	Bronchoalveolar lavage fluid
BBB	Blood–brain barrier
CAT	Catalase
COX-2	Cyclooxygenase-2
CYP450	Cytochrome P450
DCs	Dendritic cells
ECM	Extracellular matrix
FACS	Fluorescence-activated cell sorting
FLEX	Flexibility
GI	Gastrointestinal
GSH	Glutathione
HPLC	High-performance liquid chromatography
IFN	Interferon
IgE	Immunoglobulin E
IL	Interleukin
INSATU	Unsaturation
INSOLU	Insolubility
IUPAC	International Union of Pure and Applied Chemistry
JNK	C-Jun N-terminal kinase
LIPO	Lipophilicity
LOD	Limit of detection
LOQ	Limit of quantification
LPS	Lipopolysaccharide
MDA	Malondialdehyde
*m*/*z*	Mass-to-charge ratio
MMP	Matrix metalloproteinase
MAPK	Mitogen-activated protein kinase
MyD88	Myeloid differentiation primary response 88
NF-κB	Nuclear factor kappa B
OVA	Ovalbumin
PAINS	Pan Assay Interference Compounds
PBS	Phosphate-buffered saline
P-gp	P-glycoprotein
PI3K	Phosphoinositide 3-kinase
POLAR	polarity
ROS	Reactive oxygen species
RSD	Relative standard deviation
RT	Retention time
SD	Standard deviation
SMILES	Simplified molecular input line entry system
SOD	Superoxide dismutase
Smad	Suppressor of mothers against the decapentaplegic
TGF-β	Transforming growth factor beta
Th1	T helper type 1
Th2	T helper type 2
TNF-α	Tumor necrosis factor alpha
TPSA	Topological polar surface area
UPLC-QTOF-MS/MS	Ultra performance liquid chromatography-quadrupole time-of-flight tandem-mass spectrometry
WBCs	White blood cells
