# Supplementary material for: Ameliorating Effects of Phlomis umbrosa Turcz. Root in Ovalbumin-Induced Allergic Asthma: Modulation of IL-33-Mediated Inflammation and TGF-β/Smad-Dependent Fibrosis
Source: Antioxidants (Basel). 2026 Mar 27;15(4):420. doi: 10.3390/antiox15040420 (PMC13114087; doi:10.3390/antiox15040420)
Supplement: Supplementary file 1 [file antioxidants-15-00420-s001.zip › Supplementary Material.pdf]

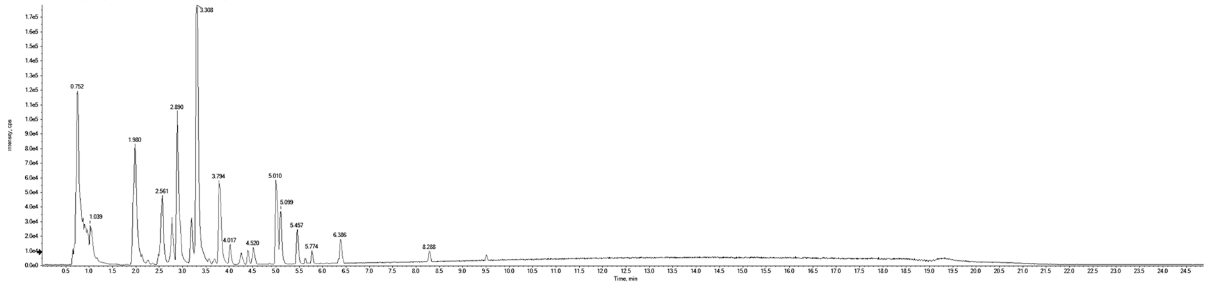

(**a**)

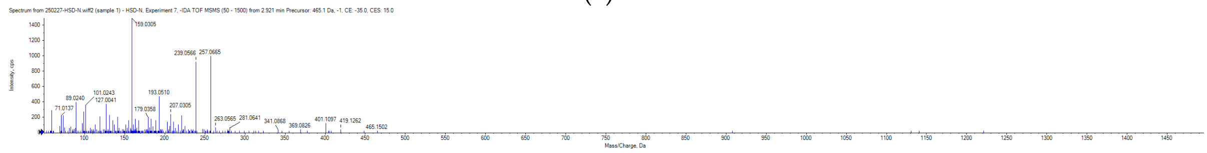

(**b**)

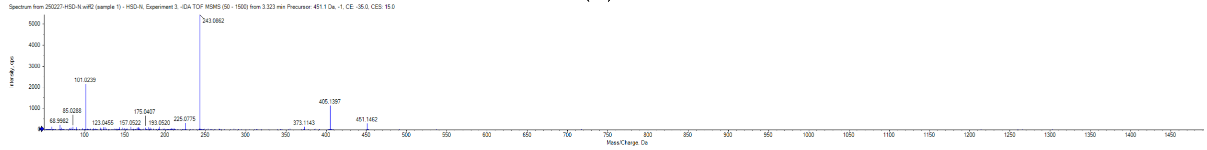

(**c**)

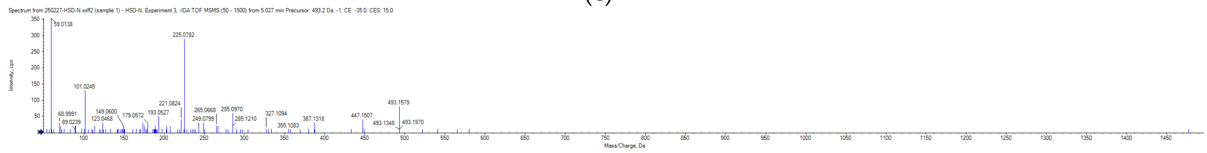

(**d**)

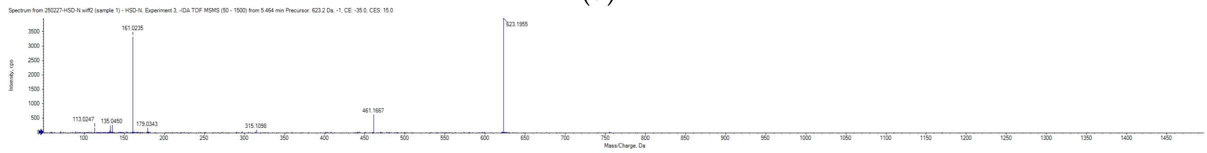

(**e**)

Figure S1. The ultra performance liquid chromatography-quadrupole time-of-flight tandem-mass spectrometry (UPLC-QTOF-MS/MS) chromatograms of the 20% ethanolic extract of *Phlomis umbrosa* Turcz. (EPT) (**a**). (**b-e**) Fragment ion peaks from sesamoside (**b**), shanzhiside methyl ester (**c**), 8-O-acetyl shanzhiside methyl ester (**d**), and isoacteoside (**e**).

Table S1. Details of antibodies utilized in this study.

Antibody	Catalog No.	Manufacturer
APC Rat Anti-Mouse CD4	553051	BD Biosciences (Franklin Lakes, NJ, USA)
BV421 Rat Anti-Mouse interferon (IFN)-γ	563376	
BV786 Hamster Anti-Mouse CD3e	564379	
PE Rat Anti-Mouse Interleukin (IL)-4	562044	
PerCP-Cy5.5-conjugated CD8a	100734	BioLegend (San Diego, CA, USA)
Anti-mouse IgG	AP124P	Millipore (Burlington, MA, USA)
Anti-rabbit IgG	7074	Cell Signaling Tech (Danvers, MA, USA)
Phospho-suppressor of mothers against decapentaplegic (p-Smad)-2	#3108	
β-actin	sc-69879	Santa Cruz Biotech (Dallas, TX, USA)
B-cell leukemia/lymphoma 2 (BCl-2)	sc-7382	
BCl-2 associated X (BAX)	sc-7480	
Caspase-3	sc-56053	
Cyclooxygenase-2 (COX-2)	sc-376861	
IL-1β	sc-515598	
IL-13	sc-393365	
IL-33	sc-517600	
IL-5	sc-398334	
Matrix metalloproteinase (MMP)-2	sc-13595	
MMP-9	sc-13520	
Myeloid differentiation primary response 88 (MyD88)	sc-74532	
Phospho-protein kinase B (p-Akt)	sc-393887	
Phospho-c-Jun N-terminal kinase (p-JNK)	sc-6254	
Phospho-nuclear factor kappa B (p-NF-κB)	sc-136548	
p-Smad-3	sc-517575	
Transforming growth factor beta (TGF-β)1	sc-130348	
Tumor necrosis factor alpha (TNF-α)	sc-33639	

Table S2. Identification of main compounds in the 20% ethanolic extract of *Phlomis umbrosa* Turcz. (EPT) using ultra performance liquid chromatography-quadrupole time-of-flight tandem-mass spectrometry (UPLC-QTOF-MS/MS) analysis.

No.	Retention time (min)	Compound	Parent ion (*m*/*z*)	Fragment ion (*m*/*z*)
1	2.890	Sesamoside	465	257, 239, 193, 159
2	3.308	Shanzhiside methyl ester	451	405, 243, 101
3	5.010	8-O-acetyl shanzhiside methyl ester	493	225, 101
4	5.457	Isoacteoside	623	461, 161

Table S3. International Union of Pure and Applied Chemistry (IUPAC) names of the major phytochemicals identified in the 20% ethanolic extract of *Phlomis umbrosa* Turcz. (EPT).

Compound	IUPAC Name
Sesamoside	methyl (1S,2R,4S,5R,6R,10S)-5,6-dihydroxy-2-methyl-10-[(2S,3R,4S,5S,6R)-3,4,5-trihydroxy-6-(hydroxymethyl)oxan-2-yl]oxy-3,9-dioxatricyclo[4.4.0.02,4]dec-7-ene-7-carboxylate
Shanzhiside methyl ester	methyl (1S,4aS,5R,7S,7aS)-5,7-dihydroxy-7-methyl-1-[(2S,3R,4S,5S,6R)-3,4,5-trihydroxy-6-(hydroxymethyl)oxan-2-yl]oxy-4a,5,6,7a-tetrahydro-1H-cyclopenta[c]pyran-4-carboxylate
8-O-acetyl shanzhiside methyl ester	methyl (1S,4aS,5R,7S,7aS)-7-acetyloxy-5-hydroxy-7-methyl-1-[(2S,3R,4S,5S,6R)-3,4,5-trihydroxy-6-(hydroxymethyl)oxan-2-yl]oxy-4a,5,6,7a-tetrahydro-1H-cyclopenta[c]pyran-4-carboxylate
Isoacteoside	[(2R,3R,4S,5R,6R)-6-[2-(3,4-dihydroxyphenyl)ethoxy]-3,5-dihydroxy-4-[(2S,3R,4R,5R,6S)-3,4,5-trihydroxy-6-methyloxan-2-yl]oxyoxan-2-yl]methyl (E)-3-(3,4-dihydroxyphenyl)prop-2-enoate
